# Effect of Ginseng (*Panax ginseng*) Berry EtOAc Fraction on Cognitive Impairment in C57BL/6 Mice under High-Fat Diet Inducement

**DOI:** 10.1155/2015/316527

**Published:** 2015-06-15

**Authors:** Chang Hyeon Park, Seon Kyeong Park, Tae Wan Seung, Dong Eun Jin, Tianjiao Guo, Ho Jin Heo

**Affiliations:** Division of Applied Life Science, Institute of Agriculture and Life Science, Gyeongsang National University, Jinju 660701, Republic of Korea

## Abstract

High-fat diet-induced obesity leads to type 2 diabetes. Recently, there has been growing apprehension about diabetes-associated cognitive impairment (DACM). The effect of ginseng (*Panax ginseng*) berry ethyl acetate fraction (GBEF) on mice with high-fat diet-induced cognitive impairment was investigated to confirm its physiological function. C57BL/6 mice were fed a high-fat diet for 5 weeks and then a high-fat diet with GBEF (20 and 50 mg/kg of body weight) for 4 weeks. After three *in vivo* behavioral tests (Y-maze, passive avoidance, and Morris water maze tests), blood samples were collected from the postcaval vein for biochemical analysis, and whole brains were prepared for an *ex vivo* test. A method based on ultra-performance liquid chromatography (UPLC) accurate-mass quadrupole time-of-flight mass spectrometry (Q-TOF/MS) was used to determine major ginsenosides. GBEF decreased the fasting blood glucose levels of high-fat diet-induced diabetes mellitus (DM) mice and improved hyperglycemia. Cognitive behavior tests were examined after setting up the DM mice. The *in vivo* experiments showed that mice treated with GBEF exhibited more improved cognitive behavior than DM mice. In addition, GBEF effectively inhibited the acetylcholinesterase (AChE) activity and malondialdehyde (MDA) levels of DM mice brain tissues. Q-TOF UPLC/MS analyses of GBEF showed that ginsenoside Re was the major ginsenoside.

## 1. Introduction

Metabolic dysfunction and obesity induced by a high-fat diet are remarkably related to learning and memory impairment [1–3]. The obesity is involved in a risk of developing insulin resistance and type 2 diabetes. In obesity, adipose tissue produces increased amounts of glycerol, proinflammatory cytokines, nonesterified fatty acids, and hormones that develop to insulin resistance. When insulin resistance is accompanied by pancreatic gland dysfunction, it leads to type 2 diabetes [2].

Recently, there has been growing apprehension about the complications of diabetes, especially diabetes-associated cognitive impairment (DACM) [4]. However, the diabetic cognitive impairments mechanisms are not completely understood. In particular, high-fat diets induce cognitive decline; one possible explanation for this relates to the proved fact that high-fat diets accompany with developed insulin resistance and attenuated glucose uptake in the brain. Impairment of glucose regulation is a significant factor in the high-fat induced cognitive defects [5]. Chronic disruption of glucose metabolism may affect cerebral blood flow, neurotransmitter metabolism, blood-brain barrier (BBB), and microvascular function, leading to memory dysfunction [6]. In addition, direct glucose toxicity in neurons leads to production of reactive species [7]. Oxidative stress may contribute to the diabetes complications [8], including DACM. Diabetes was found to induce an increase in malondialdehyde (MDA) and nitric oxide (NO) levels and mitochondrial nitric oxide synthase expression, whereas manganese superoxide dismutase content and glutathione (GSH) peroxidase activity were significantly decreased. Oxidative stress due to insults such as hyperoxia, ischemia, and metabolic disorder-induced brain damage is one of the biggest causes of neuronal injury and death in the brain [3] and results in long-term complications, morphological abnormalities, and cognitive decline [9]. Meanwhile, excessive energy intake impairs the structure and function of the hippocampus, including synaptic plasticity and neurogenesis, which are associated with cognition [10]. However, evidence of the connection between high-fat-diet-induced diabetes and cognitive impairment has not been concretely established.

Ginseng (*Panax ginseng*) is known as a medicinal herb and has been used for thousands of years. Extracts of ginseng roots, leaves, and berries have antihyperglycemic effects in type 1 and 2 diabetes animal models [11]. The active physiological compounds of ginseng are considered to be various ginsenosides. Different parts of ginseng (the root, leaf, and berry) include distinct ginsenoside compositions [12], and these parts may exhibit various physiological activities. The ginseng root is used as a traditional medicine. However, ginseng berry extract reportedly (having a higher total ginsenoside content than root extract) showed more effective antihyperglycemic activity than ginseng root extract [13].

However, no study has investigated whether ginseng berry has a protective effect against high-fat diet-induced cognitive impairment. Therefore, our study investigated the protective effects of ginseng berry on cognitive impairment caused by high-fat diet–induced diabetes in mice and identified the major ginsenosides of the ginseng berry through Q-TOF UPLC/MS.

## 2. Materials and Methods

### 2.1. Plant Materials and Extraction

Ginseng (*Panax ginseng*) berry (GB) was purchased from Guemsan in Republic of Korea (July 2014) and was authenticated by Institute of Agriculture and Life Sciences, Gyeongsang National University. The GB was dried using dry oven at 60°C for 24 hr and ground (powder type). The 50 g of GB powder was extracted with 100 mL of 80% ethanol at 40°C for 2 h, filtered through Whatman No. 2 filter paper (Whatman International Limited, Kent, UK), and evaporated using a vacuum rotary evaporator (N-N series; Eyela Co., Tokyo, Japan). The extract was resuspended in 300 mL of distilled water and separated consecutively with 300 mL of* n*-hexane, chloroform, and ethyl acetate (EtOAc). The EtOAc fraction was evaporated using a vacuum rotary evaporator at 40°C, lyophilized. The lyophilized GB EtOAc fraction was stored at −20°C until use.

### 2.2. Animals

C57BL/6 mice (male, 4-weeks old) were purchased from Samtako (Osan, Korea). These mice were divided into two per cage, and a room maintained with a 12 h light-dark cycle, 55% humidity, and 22 ± 2°C. All animal procedures were approved by the Institutional Animal Care and Use Committee (IACUC) of Gyeongsang National University (certificate: GNU-120409-M0009) and performed in accordance with the Policy of the Ethical Committee of Ministry of Health and Welfare, Korea. Lyophilized ginseng berry EtOAc fraction was mixed in drinking water (20 and 50 mg/kg of body weight) and administered orally a day for 4 weeks. Mice were divided into four groups. Group I mice were fed with normal diet for 9 weeks (control) (*n* = 8), group II mice were fed with high-fat diet (HFD) for 9 weeks (*n* = 8), group III mice were fed with high-fat diet for 5 weeks and then high-fat diet with ginseng berry EtOAc fraction (20 mg/kg, GB 20) for 4 weeks (*n* = 8), and group IV mice were fed with high-fat diet for 5 weeks and then high-fat diet with ginseng berry EtOAc fraction (50 mg/kg, GB 50) for 4 weeks (*n* = 8). Daily food intake and weekly body weight were measured.

### 2.3. Behavioral Tests with Mice

The Y-maze consisted of black-painted plastic with three arms (33 cm long, 15 cm high, and 10 cm wide, resp.). Each mouse was placed at the end of one arm and allowed free move in three arms for 8 min. The series of arm entries was recorded using video tracking system (Smart 3.0, Panlab, Barcelona, Spain), and alternation behavior was defined as entry into all three arms [14]. The spontaneous alternation behavior was calculated as a percentage of actual alternations and total alternations.

A shuttle box for passive avoidance test was divided into two chambers, illuminated zone and dark zone, and separated by a guillotine door. During the training trial, each mouse was placed in the illuminated zone. As soon as the mouse entered the dark zone, the mouse received an inescapable electric shock (0.5 mA, 3 s). After 1 day, each mouse was placed in the illuminated zone, and step-through latency time into the dark zone was evaluated (maximum time: 300 s) [15].

The Morris water maze (MWM) test consisted of a circular water pool (120 cm in diameter and 50 cm high). The pool was filled with water (20 ± 2°C) and was added white milk powders to make opaque. The pool was divided into four quadrants (W, E, S, and N zones) and, respectively, marked using a different visual cue. A white-colored platform (6 cm in diameter) was placed 1 cm below surface of water. During learning trials, mice were placed into the pool at the same starting point. The mice were freely moved for 60 s to find the hidden platform, and the found mice by oneself remained for 10 s on the platform. If the mice failed to find the platform within 60 s, the mice were placed on the platform and allowed to remain for 20 s. The mice received four consecutive daily training trials for 4 days. The time to search the platform was recorded using video tracking system. After learning trials, the hidden platform was removed, and each mouse was freely swimming for 90 s to memory retention test; the stayed time in the target quadrant (plated platform in learning trials) indicates the degree of memory about long-term learning [16].

### 2.4. Collection of Serum and Measurement of Glucose and Serum Lipids

After* in vivo* experiments, all mice were fasted for 12 h prior to sacrifice. The mice were killed by Zoletil (Virvbac SA, Carros, France) anesthesia, and blood samples were collected from postcaval vein. The blood was coagulated at room temperature for 20 min. After that, the coagulated blood by centrifugation at 10,000 g for 15 min at 4°C and supernatant (i.e., serum) was immediately frozen at −80°C until use.

The triglyceride (TG), high density lipoprotein cholesterol (HDL), total cholesterol (TC), and glucose concentrations of serum were measured by clinical chemistry analyzer (Fuji dri-chem 4000; Fujifilm Co., Tokyo, Japan). Low density lipoprotein cholesterol (LDL) was calculated by the Friedewald formula [17]: LDL (mg/dL) = total cholesterol − (HDL + TG/5). The ratio of HDL to total cholesterol (HTR, %) was calculated as HTR (%) = (HDL/TC) × 100. Fasting blood glucose level was measured from the tail vein blood using a glucose meter before* in vivo* experiments [18].

### 2.5. Preparation of Tissue Samples after* In Vivo* Experiments

For* ex vivo* biochemical studies, mice were sacrificed. Brains were removed and kept at −80°C until use. Whole brains were homogenized with 10 volumes of cold phosphate buffered saline in an ice bath.

### 2.6. Determination of* Ex Vivo* AChE Activity

The AChE enzymatic assay was conducted according to the colorimetric method using acetylthiocholine iodide as a substrate [19]. Whole brains homogenates were centrifuged 12,000 rpm for 30 min at 4°C, and the supernatants were used for AChE assay. The supernatant (5 *μ*L) was mixed with 65 *μ*L of 50 mM sodium phosphate buffer (pH 7.4) and incubated at 37°C for 15 min. After incubation, mixtures were added to an 70 *μ*L of Ellmans's reaction mixture [0.5 mM acetylthiocholine and 1 mM 5,5′-dithio-bis(2-nitrobenzoic acid) in a 50 mM sodium phosphate buffer (pH 7.4)] and incubated at 37°C for 20 min. Reading was repeated for 10 min at 2 min using microplate reader (680, Bio-Rad, Tokyo, Japan). The data were expressed as percent relative to the activity of the control group (100%).

### 2.7. Determination of* Ex Vivo* MDA Levels

The MDA level which is a measure of lipid peroxidation was determined by thiobarbituric acid (TBA) reactive substance formation in mice brain homogenates. Briefly, 160 *μ*L homogenate was mixed with 960 *μ*L of 1% (v/v) phosphoric acid and 320 *μ*L of 0.67% (w/v) TBA solution. The mixtures were then heated in the water bath at 95°C for 1 h. After cooling, the mixtures were centrifuged 600 g for 10 min to obtain the supernatant, and the supernatant was measured at 532 nm using spectrophotometer, and MDA level unit was expressed as nmole/mg of protein [15].

### 2.8. Ultra-Performance Liquid Chromatography (UPLC) Accurate-Mass Quadrupole Time-of-Flight (Q-TOF)/MS Analysis

The chromatographic separation was performed using an ultra-performance liquid chromatography (UPLC) accurate-mass quadrupole time-of-flight (Q-TOF) MS (Agilent Technologies, Santa Clara, CA, USA), and column was used to a ACQUITY UPLC BEH C_18_ column (2.1 × 100 mm, 1.7 *μ*m particle size; Waters Corp, Milford, MA, USA) with a flow rate of 0.3 mL/min and oven temperature at 40°C. A linear solvent gradient of binary mobile phase (mobile solvent A; 0.1% formic acid in distilled water; mobile solvent B, 0.1% formic acid in acetonitrile) during analysis was applied as follows: 99% A/1% B at 0 min, 99% A/1% B at 4 min, 50% A/50% B at 16 min, 50% A/50% B at 20 min, and 99% A/1% B at 24 min. The ESI-MS conditions were negative ion mode, capillary voltage (5 kV), dry gas (N_2_, 10 L/min), temperature (350°C), pressure of nebulizer (40 psi), fragmentor voltage (175 V), and mass range (100 to 1200* m/z*).

### 2.9. Statistical Analysis

Results were expressed as mean ± SD. Each experimental set was analyzed by one-way analysis of variance (ANOVA) and Duncan's multiple-range test (*p* < 0.05) using SAS software (SAS Institute Inc., Cary, NC, USA).

## 3. Results

### 3.1. Body Weight, Food Intake, Glucose Levels, and Serum Lipids

The final body weights of the GB 50 group were significantly higher than those of the high-fat diet (HFD) group (Table 1). However, the food intake of the HFD group was higher than that of the GB 50 group. The fasting glucose levels of the HFD group were also significantly higher than those of the control group. On the other hand, the fasting glucose levels of the GB 20 and GB 50 groups were decreased effectively compared with those of the HFD group (by approximately 17% and 21%, resp.).

The GB groups had lower serum triglyceride (TG), total cholesterol (TC), and low density lipoprotein cholesterol (LDL) concentrations compared with the HFD group (Table 2). Meanwhile, the GB 50 group in particular had an increased ratio of HDL to TC (approximately >29% HTR). Serum glucose levels were also examined to confirm HFD-induced diabetes mellitus; these were significantly lower in the GB groups. In particular, the serum glucose levels of the GB 50 group were lower than those of the control group.

### 3.2. Effect of the Ginseng Berry Ethyl Acetate Fraction (GBEF) on Y-Maze Test, Passive Avoidance Test, and Morris Water Maze Test

To determine the attenuating effect of the GBEF against high-fat diet-induced cognitive impairment, several behavioral tests were utilized.

**Figure 1:**
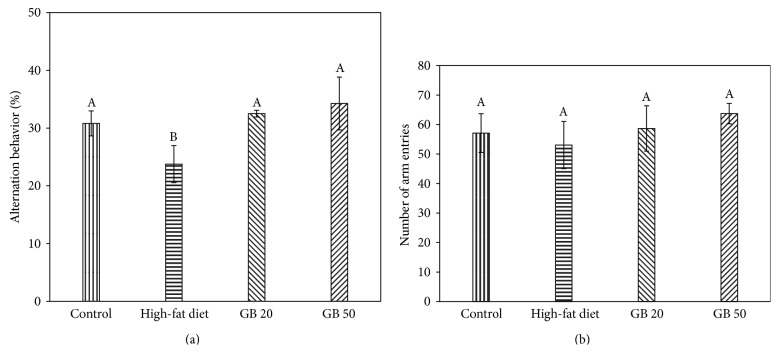
Effect of ethyl acetate fraction from ginseng berry on spontaneous alternation behavior. Alternation behavior (a), number of arm entries (b). Contorl; normal diet, High-fat diet, GB 20; high-fat diet with GBEF 20 mg/kg of body weight, GB 50; high-fat diet with GBEF 50 mg/kg of body weight. Results shown are mean ± SD (*n* = 8). Data were statistically considered at *p* < 0.05, and different small letters represent statistical difference.

The Y-maze test is a hippocampal neuron-dependent task that investigates spatial working memory. This maze test is different from the passive avoidance test in the fact that the Y-maze test is based on the congenital inclination of mice to explore a new experience rather than learn a new behavior [20]. The GBEF ameliorated high-fat diet-induced cognitive impairment. The high-fat diet-fed mice showed impaired spatial working memory compared with the control group (Figure 1(a)). Treatment with the GBEF improved spontaneous alternation behavior in the high-fat diet-fed mice. In addition, no statistical differences were found between the numbers of arm entries in all experimental groups, which indicated that general locomotor activity was not influenced by a high-fat diet (Figure 1(b)).

**Figure 2:**
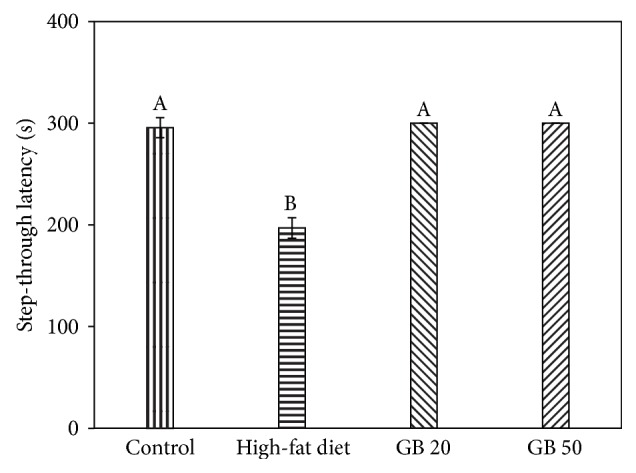
Effect of ethyl acetate fraction from ginseng berry on passive avoidance test. Contorl; normal diet, High-fat diet, GB 20; high-fat diet with GBEF 20 mg/kg of body weight, GB 50; high-fat diet with GBEF 50 mg/kg of body weight. Results shown are mean ± SD (*n* = 8). Data were statistically considered at *p* < 0.05, and different small letters represent statistical difference.

The passive avoidance test is an amygdala-dependent task that examines the ability of mice to learn and memorize an associative regulation. It has been related to long-term memory. N-Methyl-D-aspartic acid (NMDA) receptors in the brain are involved in the arrangement of post achieving memory in the amygdala and hippocampus [21]. In the passive avoidance test, the high-fat diet-fed mice showed significantly shorter latency times during the testing trials, with an approximate 33% decrease in step-through latency compared with the control group. However, the GB 20 and GB 50 groups exhibited improved high-fat diet-induced learning and memory impairment, and the results were similar to those of the control group (Figure 2).

**Figure 3:**
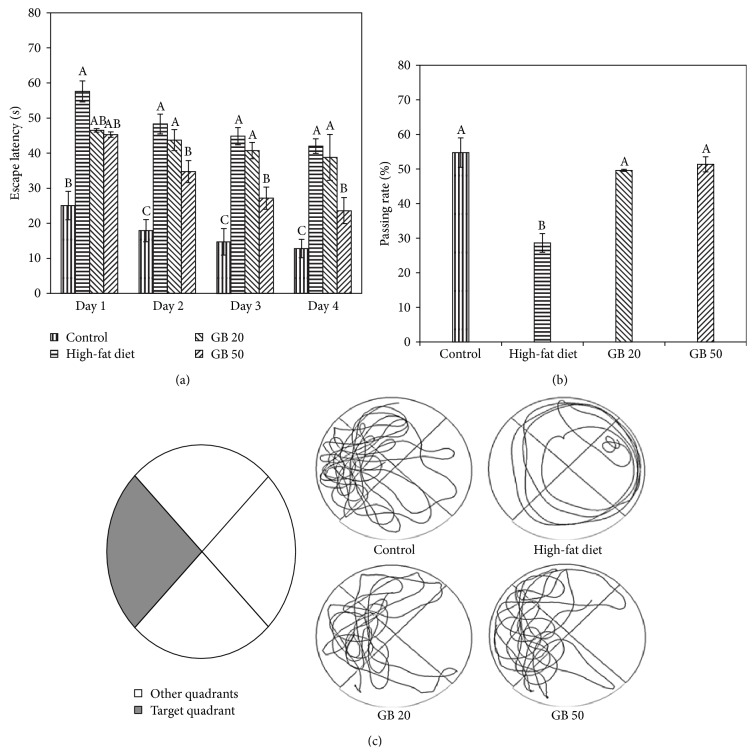
Effect of ethyl acetate fraction from ginseng berry on the Morris water maze test. Escape latency in the training trial (a), passing rate in the probe trial (b), and path tracing of each group in the probe trial (c). Contorl; normal diet, High-fat diet, GB 20; high-fat diet with GBEF 20 mg/kg of body weight, GB 50; high-fat diet with GBEF 50 mg/kg of body weight. Results shown are mean ± SD (*n* = 8). Data were statistically considered at *p* < 0.05, and different small letters represent statistical difference.

Long-term learning and memory functions were examined using the Morris water maze test, and the escape latencies from days 1 to 4 were statistically measured. The mean escape latency for the trained mice decreased over the course of the 16 learning trials in all groups. However, the GB groups showed lower escape latencies on days 1, 2, 3, and 4 during the training trials compared with the HFD group. In particular, the GB 50 group had significantly decreased escape latencies from days 1 to 4 of the training trials (Figure 3(a)). In the probe trial, which measured how well the mice had learned and consolidated the platform location during the 4 days of training, the path tracings of the mice showed that the mice in the control group circled relatively more often around the platform zone (54%), whereas the mice in the high-fat diet-fed group circled around all zones (28%) (Figure 3(b)). The swim patterns also showed that the GBEF-treated groups had clear search patterns over the area where the hidden platform used to be, whereas the high-fat diet-fed mice showed more random search patterns (Figure 3(c)). The time spent in the target quadrant (platform zone) was significantly lower in the high-fat diet-fed group compared with all the groups. Consequently, the GBEF-treated groups spent more time in the target quadrant than the high-fat diet-fed group in the probe test.

### 3.3. Effect of the Ginseng Berry Ethyl Acetate Fraction (GBEF) on AChE Activity and Lipid Peroxidation Levels in High-Fat Diet-Fed Mice Brains

**Figure 4:**
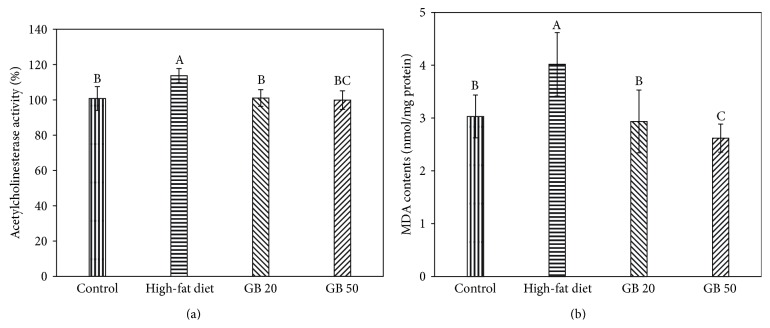
Effect of ethyl acetate fraction from ginseng berry on AChE activity (a) and MDA level (b) from high-fat diet mice brain homogenates. Contorl; normal diet, High-fat diet, GB 20; high-fat diet with GBEF 20 mg/kg of body weight, GB 50; high-fat diet with GBEF 50 mg/kg of body weight. Results shown are mean ± SD (*n* = 8). Data were statistically considered at *p* < 0.05, and different small letters represent statistical difference.

After the* in vivo* experiments, the AChE activity in the mouse brains was determined to confirm the cholinergic effect of the GBEF treatment. The high-fat diet-fed group showed increased AChE activity (113%), whereas the GB 20 and GB 50 groups showed relatively inhibited AChE activity (101% and 100%, resp.) (Figure 4(a)).

We also examined whether the GBEF could inhibit high-fat diet-induced lipid peroxidation by measuring the levels of MDA in the brain homogenates. The levels of MDA were increased (4 nmol/mg protein) in the high-fat diet-fed group compared with those of the control group (3 nmol/mg protein). The MDA levels of the GB groups significantly decreased in a dose-dependent manner (Figure 4(b)). The GB 50 group presented lower MDA contents (2.6 nmol/mg protein) than those of the control group.

### 3.4. UPLC Q-TOF/MS Analysis of Ginsenosides in the Ginseng Berry Ethyl Acetate Fraction (GBEF)

**Figure 5:**
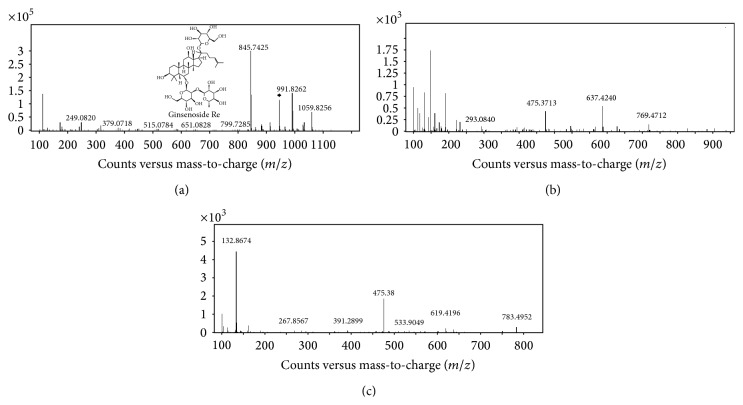
Q-TOF UPLC/MS spectra in negative ion mode and chemical structures of ginsenoside Re. MS scan data for ginsenoside Re (a). MS^2^ patterns were obtained with indicated collision energies (CE) 40 eV (b) and CE 50 eV (c).

The ginsenosides in GBEF were analyzed using UPLC Q-TOF/MS for retention time, UV-VIS spectrum, MS/MS scanning for mass fragmentation, and the comparison of MS spectra obtained from the Q-TOF LC/MS (MS/MS) fragmented spectra data from a previous report [22]. The ESI-MS spectra of ginsenoside Re were exhibited on negative ion mode [M−H]^−^ at* m/z* 945 and adduct ions [M+formic acid]^−^ at* m/z* 991 (Figure 5(a)). The MS/MS spectra were obtained by collision-induced dissociation (CID) from negative ion mode. The parent ion of ginsenoside Re was* m/z* 945, and the main five fragment ions were* m/z* 799, 783, 637, 619, and 475 (Figure 5). The fragment ion* m/z* 799 as loss of one rhamnose molecule (*m/z* 146) and the* m/z* 783 fragment ion as loss of one glucose molecule (*m/z* 162) were, respectively, examined. These losses of different-formed sugar molecules indicated two different terminal sites of the glycosidic moieties in their chemical structure. The* m/z* 637 fragment ion (loss of both rhamnose and glucose molecules), the* m/z* 619 fragment ion (loss of each one of the rhamnose, glucose, and H_2_O molecules) and the* m/z* 475 fragment ion (loss of all linked glucose molecules, 20(*S*)-protopanaxatriol aglycon form) were examined. Therefore, the MS/MS spectra were analyzed by the observation of the two main fragment ions produced such as the loss of the saccharide molecules or the H_2_O molecule. Therefore, these data showed that the major ginsenoside of GBEF is the Re type.

## 4. Discussion

In the present study, we examined the effect of ginseng berry on the biochemical functions of high-fat diet-induced type 2 diabetes and behavioral defects in mice. High-fat diet-associated diabetes caused cognitive impairment, which were accompanied by increased AChE activity and MDA levels in mice brains. Treatment with GBEF remarkably and dose-dependently improved cognitive impairment through cholinergic enzyme dysfunction and antioxidant activity.

The significant hypoglycemic effect of GBEF in high-fat diet-associated cognitively impaired mice was determined, and the glucose levels (fasting and serum) after 4 weeks of treatment with GB 20 (GBEF 20 mg/kg of body weight) and GB 50 (GBEF 50 mg/kg) were decreased effectively. In addition, GBEF treatment significantly improved weight loss and TG, TC, LDL, and HTR (%) levels, most likely as a consequence of improved diabetic symptoms. Weight loss, polyphagia, hypercholesteremia, hyperlipidemia, and hyperglycemia are recognized as typical diabetic signs [23]. Therefore, the results of the biochemical analysis of mice serum showed that the increase in TC, LDL, TG, and HTR (%) caused by a high-fat diet was due to the imbalance in lipid metabolism. High-fat diet-associated obesity can impair neuronal insulin receptors and glucose metabolism, which is considered to be an important mechanism of neurodegeneration, such as Alzheimer's disease [24]. In a previous study, ginseng berry extract improved blood glucose levels, ameliorated insulin sensitivity, lowered cholesterol, and decreased body weight in type 2 diabetic mice [25]. Chronic treatment with ginseng berry ameliorated hyperglycemia, lowered lipid peroxidation, and increased the reduced glutathione levels of diabetic mice [26]. Therefore, chronic treatment with ginseng berry could ameliorate the cognitive impairment caused by diabetes and decrease oxidative stress in type 2 diabetic mice. Our results suggest that ginseng berry may ameliorate diabetes-associated cognitive impairment by decreasing blood glucose levels and improving serum lipid balance and antioxidant activity.

The Y-maze test evaluated short-term spatial recognition, and the passive avoidance test examined the prevention of retention and abnormality recovery [27]. The Y-maze test is a behavioral test for measuring the willingness of mice to explore new environments. Mice typically prefer to investigate a new arm of a maze rather than returning to one that has been previously visited. The passive avoidance test is a fear-motivated test used to investigate learning and memory in mice [28]. In this test, mice learn to avoid an environment in which an aversive stimulus (such as an electric foot shock) has been previously received. The high-fat diet-fed group immediately showed working memory impairment in the Y-maze test, and their alternation behavior scores were lower than those of the control and GB groups. The high-fat diet-fed group also showed a significant reduction in step-through latency compared with the control group, whereas the high-fat diet–induced learning and memory decline of the mice in the passive avoidance test was remarkably attenuated in the GB group. The ameliorating effect of GBEF in high-fat diet-associated cognitive impairment might be related to its antihyperglycemic effect.

The Morris water maze task is based on the reward principle and is a test of spatial learning and memory in which mice must depend on distal cues to navigate from start locations around the perimeter of a water poll to find a hidden platform. Spatial learning and memory are evaluated through training trials, and reference memory is confirmed by the affinity for the platform area when the platform is absent [29]. Mice dislike swimming and typically attempt to escape from the water. Eventually, the mice searched for a hidden platform. The high-fat diet-fed group performance in the Morris water maze test was impaired, as evidenced by the higher escape latency times (Figure 3). The GBEF-treated groups generally showed shorter escape latencies than the high-fat diet-fed group. Treatment with GBEF significantly contributed to memory acquisition in learning the location of the platform, demonstrating an enhancement of spatial learning and memory in the mice. The GBEF-treated groups displayed shorter escape latency times than the high-fat diet-fed group during the first trial of each day (Figure 3(a)). The effect on memory retention was determined by the assessment of reference memory using a probe test (Figure 3(b)). Finally, the results of the training trial and probe test suggested that GBEF might affect the memory acquisition of high-fat diet-fed mice.

Acetylcholine, a cholinergic neurotransmitter in the brain-nerve system, is decomposed by AChE; thus, immoderate AChE release might disrupt cognitive function [30]. Amyloid beta-protein (A*β*) induced oxidative stress contributes to neuronal lipid peroxidation, protein oxidation, DNA oxidation, and increased Ca^2+^ influx into the cell, mainly through sensitive voltage-gated Ca^2+^ channels, and these results were related to the increase of AChE activity. Therefore, high-fat diet-induced oxidative stress caused cognitive defects according to the increase of AChE activity [30]. In this regard, the high-fat diet-fed group showed increased AChE activity compared with the control group, and GBEF-treated groups showed decreased activity compared with the high-fat diet-fed group.

Oxidative stress is accompanied with the pathogenesis of diabetes and its side effects. A present study showed that increased levels of oxygen free radicals and the decreased effectiveness of the antioxidant system made neurons and astrocytes more vulnerable to damage in high-fat diet-induced diabetes [31]. Increase in free radicals, which are formed in diabetes by glucose oxidation, protein glycation, and the subsequent degradation of glycated proteins, are involved in diabetes-associated cognitive impairment, and their overproduction may be considered one of the most important factors for insulin resistance [32]. Reactive oxygen species and the sensitivity of brain tissue to oxidative stress have been well researched. In particular, polyunsaturated fatty acids are highly sensitive to oxidative degeneration because of their double bonds. Lipid peroxidation in brain tissue indicates degeneration of the neuronal membrane. Malondialdehyde (MDA) has been used as an indicator of lipid peroxidation of cell membranes and free radical generation in the brain homogenates [33]; thus, elevated MDA levels indicate neuronal degeneration. The high-fat diet-fed group showed an increase in MDA levels in mice brains, whereas those in the GBEF treatment group (GB 20 and GB 50) were significantly decreased compared with those of the high-fat diet-fed group. These observations suggest that GBEF may have antiamnesic effects because of its significant antioxidant activity in high-fat diet-fed mice.

A previous study found that ginsenoside Re and Rg1 were the most abundant ginsenosides in roots and berries. However, ginseng berries have more total ginsenosides and the ginsenoside Re content of berries was 4–6 times higher than that of roots. The amounts of the five ginsenosides in the berries were Re > Rc = Rg1 = Rb1 = Rd and in the roots Re > Rg1 > Rb1 > Rc > Rd [34]. In the present study, the ginsenosides of GBEF were identified by UPLC Q-TOF/MS. The parent ion of ginsenoside Re was at* m/z* 945 [M−H]^−^, and the adduct ions were at* m/z* 991 [M−H+formic acid]^−^. In general, adduct ions depend on the mobile phase modifier. Furthermore, five main fragment ions at* m/z* 799 [M−H-Rha]^−^, 783 [M−H-Glc]^−^, 637 [M−H-Rha-Glc]^−^, 619 [M−H-Rha-Glc-H_2_O]^−^, and 475 [M−H-Rha-2Glc]^−^ were observed. Ginsenoside Re attenuates significant hyperglycemia and could ameliorate the imbalanced oxidative stress in the kidneys and eyes of diabetic mice [35]. In addition, ginsenoside Re demonstrates conclusive activity towards hypertriglyceridemia and hypercholesterolemia in diabetes [36].

Hyperglycemia reduces the effectiveness of antioxidant systems and concurrently increases free radical generation. Augmented glucose levels and oxidative stress occur together in diabetes [37]. Hyperglycemia is a critical determinant of cognitive impairment in type 2 diabetes patients [38], suggesting a requirement of glycemic control for the improvement of diabetes-induced cognitive impairment. Consequently, the antihyperglycemic effect of ginsenoside Re in GBEF might be a pivotal factor for improving high-fat diet-induced cognitive impairment.

## 5. Conclusion

In conclusion, long-term treatment with GBEF ameliorated cognitive defects due to cholinergic activity and reduced oxidative stress in the high-fat diet-induced type 2 diabetic mouse model. Consequently, the promising antioxidant and antihyperglycemic effects of ginseng berry with ginsenoside Re may open new avenues in the treatment of diabetes-induced cognitive impairment.

## Figures and Tables

**Table 1:** Effect of ethyl acetate fraction from ginseng berry on body weight, food intake, and fasting glucose level in high-fat diet-induced diabetic mice.

Treatment	Body weight (g)	Food intake (g/day)	Fasting glucose (mg/dL)
Control	33.25 ± 1.25^c^	2.36 ± 4.22^b^	241.67 ± 6.11^d^
High-fat diet	44.55 ± 4.50^b^	2.78 ± 1.46^a^	381.67 ± 8.96^a^
GB 20^(1)^	45.25 ± 2.62^b^	2.44 ± 1.70^b^	319 ± 10.54^b^
GB 50^(2)^	47.33 ± 0.57^a^	2.16 ± 1.68^c^	304.33 ± 7.64^c^

Results shown are mean ± SD (*n* = 8). Data were statistically considered at *p* < 0.05, and different small letters represent statistical difference.

^(1)^GB 20: high-fat diet + ginseng berry EtOAc fraction (20 mg/kg of body weight).

^(2)^GB 50: high-fat diet + ginseng berry EtOAc fraction (50 mg/kg of body weight).

**Table 2:** Effect of ethyl acetate fraction from ginseng berry on serum analysis in high-fat diet-induced diabetic mice.

Treatment	TG (mg/dL)	TC (mg/dL)	LDL^(1)^ (mg/dL)	Glucose (mg/dL)	HTR^(2)^ (%)
Control	58.33 ± 7.23^b^	116.33 ± 1.53^c^	59.44 ± 2.31^c^	294 ± 6.56^b^	39.25 ± 2.34^bc^
High-fat diet	81.33 ± 8.96^a^	187.66 ± 7.37^a^	101.16 ± 1.23^a^	412 ± 2.65^a^	37.47 ± 1.45^c^
GB 20^(3)^	67 ± 9.85^ab^	145.33 ± 5.03^b^	68.81 ± 2.4^b^	304.33 ± 7.51^b^	43 ± 2.16^b^
GB 50^(4)^	63 ± 3^b^	138 ± 7.21^b^	58.83 ± 1.45^c^	240.33 ± 10.02^c^	48.31 ± 1.34^a^

Results shown are mean ± SD (*n* = 8). Data were statistically considered at *p* < 0.05, and different small letters represent statistical difference.

^(1)^LDL (mg/dL) = TC − (HDL + TG/5).

^(2)^HTR (%) = (HDL/TC) × 100.

^(3)^GB 20: high-fat diet + ginseng berry EtOAc fraction (20 mg/kg of body weight).

^(4)^GB 50: high-fat diet + ginseng berry EtOAc fraction (50 mg/kg of body weight).
